# The outcast of medicine: metals in medicine--from traditional mineral medicine to metallodrugs

**DOI:** 10.3389/fphar.2025.1542560

**Published:** 2025-04-07

**Authors:** Donghan Bai, Michal Nowak, Dajun Lu, Qiaochu Wang, Martin Fitzgerald, Hui Zhang, Remy MacDonald, Ziwen Xu, Lu Luo

**Affiliations:** ^1^ Institute of Chinese Materia Medica, China Academy of Chinese Medical Sciences, Beijing, China; ^2^ Faculty of Medicine, Poznan University of Medical Sciences, Poznan, Poland; ^3^ Cell Biology Program, Memorial Sloan Kettering Cancer Center, New York, NY, United States; ^4^ Department of Biochemistry and Molecular and Cellular Biology, Georgetown University, Washington, DC, United States; ^5^ Ashdale Clinic, Cork, Ireland; ^6^ Institute of Traditional Chinese Medicine, European University of Chinese Medicine, Horsens, Denmark; ^7^ Department of Statistics, George Mason University, Virginia, VA, United States; ^8^ Department of Nursing, The University of Melbourne, Parkville, VIC, Australia

**Keywords:** metallodrug, traditional Chinese medicine, inorganic medicine, mineral medicine, anticancer, integrative medicine

## Abstract

Metals have long held a significant role in the human body and have been utilized as mineral medicines for thousands of years. The modern advancement of metals in pharmacology, particularly as metallodrugs, has become crucial in disease treatment. As the machanism of metallodurgsare increasingly uncovered, some metallodrugs are already approved by FDA and widely used in treating antitumor, antidiabetes, and antibacterial. Therefore, a thorough understanding of metallodrug development is essential for advancing future study. This review offers an in-depth examination of the evolution of mineral medicines and the applications of metallodrugs within contemporary medicine. We specifically aim to summarize the historical trajectory of metals and mineral medicines in Traditional Chinese Mineral Medicine by analyzing key historical texts and representative mineral medicines. Additionally, we discuss recent advancements in understanding metallodrugs’ mechanisms, such as protein interactions, enzyme inhibition, DNA interactions, reactive oxygen species (ROS) generation, and cellular structure targeting. Furthermore, we address the challenges in metallodrug development and propose potential solutions. Lastly, we outline future directions for metallodrugs to enhance their efficacy and effectiveness. The progression of metallodrugs has broadened their applications and contributed significantly to patient health, creating good healthcare solutions for the global population.

## 1 Introduction

Throughout history, metals have played both therapeutic and toxic roles in medicine. It is well understood that many metals are essential for the human organism and have been applied in medicine for thousands of years. From ancient practices that utilized minerals for healing to the sophisticated metallodrugs of modern pharmacology, the journey of metals in medicine is a testament to their complexity and significance.

In traditional medicine, various cultures recognized the healing properties of metals and minerals. The documented therapeutic application of metals in humans is as old as civilization genesis. From Ancient India, Egypt, to the Roman Empire, metallic copper was employed to sterilize water and prevent the spread of diseases (AHMAD et al., 1995). Ancient Egyptians and Aztecs also used copper sulfate and copper oxide to sterilize wounds and treat skin diseases (Dollwet et al., 1985). Similarly, in India, the practice of Rasashastra in Ayurveda has long utilized processed metals, such as mercury, gold, and copper, for medicinal purposes. The elaborate detoxification techniques (shodhana) described in Rasashastra texts, aimed at reducing mercury toxicity, prefigure modern chelation strategies (Savrikar and Ravishankar, 2011). Iron oxide and iron salts were widely used in Egypt and Greece to treat hair loss and anemia, respectively (Elston, 2010). Gold and silver have also been used by ancient civilizations in Arabia, China, and Greece for medicinal purposes (Bingham and Cohrssen, 2012; Rahman and Singh, 2019). However, as our understanding of chemistry and biology has evolved, our appreciation for the duality of metal medicine has grown together (Allardycec and Dyson, 2016). While certain metals can enhance health, others—like cadmium, lead and mercury—are notorious for their toxicity and their use has been regulated (Rahman and Singh, 2019).

The transition from traditional mineral medicine to modern metallodrugs is a significant milestone in pharmacology. Metallodrugs, which include metal-containing compounds used in treatment, have gained prominence in recent decades, particularly in oncology (Andrés et al., 2024). One of the most notable examples is cisplatin, a platinum-based chemotherapeutic agent that revolutionized cancer treatment (Ghosh, 2019; Siddik, 2003). Its mechanism of action, which involves DNA binding and the induction of apoptosis in cancer cells, highlights the potential of metals as effective therapeutic agents (Bashir et al., 2023). Intriguingly, such modern innovations often resonate with traditional paradigms. For instance, arsenic trioxide (ATO)—now a frontline therapy for acute promyelocytic leukemia—directly descends from arsenic sulfide prescriptions in Traditional Chinese Mineral Medicine (TCMM), while the nanoparticulate gold in Ayurvedic Swarna Bhasma has inspired biocompatible gold-based therapies for rheumatoid arthritis (Bensky, 1992). Moreover, the development of new metallodrugs continues to expand, with ongoing research exploring a variety of metal ions, including ruthenium, gallium, and arsenic, each offering unique mechanisms of action and therapeutic possibilities (Lucaciu et al., 2022; Lee et al., 2020; Schuh et al., 2012). These advancements are not without challenges, as issues such as metal toxicity, resistance, and bioavailability remain critical areas of investigation (Anthony et al., 2020). As we reflect on the historical and contemporary roles of metals in medicine, their trajectory reveals an iterative dialogue between empirical tradition and molecular precision. Ancient systems like TCMM and Ayurveda provide more than historical footnotes—they offer clinically refined templates for modern metallodrug development. Ayurvedic formulations such as Tamra Bhasma (processed copper) demonstrate how traditional processing can enhance metal bioavailability (Wadekar et al., 2005), mirrored in TCMM’s use of calcined minerals (e.g., Mengshi) to reduce raw ore toxicity. These time-tested approaches, validated through centuries of observational practice, effectively pre-screen metal candidates and combinatorial protocols, thereby accelerating contemporary drug discovery pipelines (Bensky et al., 2015).

As we reflect on the historical and contemporary roles of metals in medicine, it becomes evident that they are neither mere outcasts nor universally embraced (Bertrand et al., 2014). Instead, they occupy a nuanced space where traditional knowledge intersects with cutting-edge science. This review aims to elucidate the potential of metals in medicine, with a focus on Traditional Chinese Mineral Medicine (TCMM), highlighting their journey from ancient practices to modern innovations, while addressing the challenges that lie ahead.

## 2 The early origins and modern evolution of mineral medicines in TCMM

### 2.1 The origins of mineral medicines in TCMM

TCMM has been an integral part of Chinese medical practices for over 2,000 years and, importantly, it has been well documented. Early texts like the *Shennong Bencao Jing* (Divine Farmer’s Materia Medica) (Yang, 1998), written between 200 BCE–200 CE (Han Dynasty), documents the use of mineral substances for therapeutic purposes. *Shennong Bencao Jing* lists several minerals, including cinnabar (mercury sulfide), realgar (arsenic sulfide), and gypsum (calcium sulfate), highlighting their roles in treating ailments such as inflammation, infections, and anxiety. This text, one of the earliest pharmacopeias, also classified medicinal substances, including minerals, into three categories: “superior,” “middle,” and “inferior” medicines (Unschuld, 1986). 18 Minerals were categorized as “superior,” which is higher than the other two categories, due to they were crucial for treating severe diseases. However, with a deep understanding of these mineral medicines, the toxicity was recognized by the physicians. Some of them were less and less used over a long period historically (Bingham and Cohrssen, 2012). The research and development never stopped cause of their powerful efficacy and widely used in many different medical conditions. These mineral medicines were believed to interact with the body’s qi (vital energy) and harmonize imbalances in the five elements theory (Wood, Fire, Earth, Metal, and Water) that underpins traditional Chinese medicine. For example, cinnabar was associated with calming the mind and treating heart-related conditions due to its connection with the Fire element (Liu et al., 1988). Together, mineral medicines, qi, and five elements theory form the backbone of traditional Chinese Medicine (TCM).

### 2.2 The morden evolution of mineral medicines of TCMM

Several historical texts have documented the evolving understanding and use of mineral medicines in TCMM. These documents provide important evidence for the evolution of mineral medicines (Boulikas et al., 2007). *Shennong Bencao Jing* listed 365 substances, of which 46 were minerals, including cinnabar, realgar, and gypsum. However, some minerals were widely used in practice beyond *Shennong Bencao Jing* described, for instance, potent treatment for ailments like fever, mental disturbances, and skin conditions (Hsu, 2001; Bensky, 1992). For example, Cold-Food Powder, which mixes fluorite, quartz, red bole clay, stalactite, and sulfur, all the compound is recorded in *Shennong Bencao Jing*, however, it was fully developed and widely used after 202–589 (Six Dynasties). Between 618 and 907 (Tang Dynasty), TCMM saw significant development, particularly in terms of classifying and expanding mineral use. Sun Simiao (?-682), a renowned physician, contributed greatly with his text, *Qian Jin Yao Fang* (Essential Formulas Worth a Thousand Pieces of Gold) (Simiao, 1982). He describes the medicinal use of various minerals, such as magnetite, used to calm the mind and improve sleep, and realgar, believed to dispel toxins (Simiao, 1982). Another one is *Taoist Alchemy*, which was composed by the Taoist alchemists. It explored the medicinal and mystical properties of minerals, seeking immortality. Minerals like cinnabar, mercury, and arsenic were incorporated into longevity elixirs, despite their potential toxicity (Needham et al., 1980). Pharmacopoeia between 960 and 1,279 (Song Dynasty), *Kaibao Bencao* (973), further refined the classification of minerals, organizing them based on properties like temperature and taste to optimize their therapeutic use (Hao and Jiang, 2015). *Bencao Gangmu* (Compendium of Materia Medica) was compiled by Li Shizhen between 1,368 and 1,644 (Ming Dynasty) (Shinzhen, 2006). This exhaustive text cataloged over 1,800 substances, including numerous minerals, more than 160 substances (Bratsos et al., 2007). It expanded the pharmacological scope of TCMM by providing detailed descriptions of mineral properties, preparations, and therapeutic applications. Especially *Bencao Gangmu* included entries on minerals such as magnetite, gypsum, and realgar, and described their preparation methods to reduce toxicity. Li’s work significantly influenced the later development of both TCMM and Western pharmacopeias (Needham et al., 1980; Shinzhen, 2006). While some of these alchemical practices led to harmful outcomes, they provided insights into the effects of minerals on the human body, laying the foundation for modern mineral pharmacology in TCM (Despeux, 2018) (Table 1).

**TABLE 1:** Representative mineral medicines in historical texts in China.

Dynasties	Representative works	Authors (last name, first name)	Number of records	Representative medicines	Outstanding achievements	Historical significance	Period characteristics
Shang	(Excavated Cultural Relics)			Cinnabar	Cinnabar used as pigment for oracle bone inscriptions	Early mineral application in recorded history	Primitive utilization of minerals
770–221 BCE (Spring and Autumn Period)	Guanzi - Di Shu	Guan, Zhong		Cinnabar	Revealed mineral symbiosis laws and indicator minerals	Embryonic concepts of genetic mineralogy	Emergence of mineralogical thought
	The Classic of Mountains and Seas	unspecified	66 animal drugs, 51 plant drugs and 2 mineral medicines were recorded	Androbus, ochre, arsenic	Earliest written record of mineral medicinal use	①Pioneering Chinese classic; ②Origin of mineral pharmacology	Integration of geography and pharmacology
221 BCE - 220 CE (Qin and Han dynasties)	Formularies for 52 Disorders	unspecified	242 drugs, of which 21 were used as mineral medicines	realgar, mercury	Deepened understanding of mineral medicines	Oldest surviving mineral medicine records	Rise of alchemy; royal pursuit of immortality boosted mineral drug research
	The Divine Husbandman’s Classic of the Materia Medica	unspecified	365 drugs, and 46 mineral medicines	Dansha/mercury	①Classified minerals into upper (18), middle (14), lower (9) grades; ②Systematized properties	World’s earliest records of mercury/arsenic-based medicines	Alchemy-driven mineral studies
	Huai Nan Wan Bi Shu	Liu an		copper (II) sulfate	①The earliest written record of the metal substitution reaction ② The first detailed introduction of mineral medicines such as dansha, mercury, lead, and zengqing as raw materials for alchemy		Early chemical experimentation
220 CE - 589 CE (Wei-Jin and the Northern and Southern Dynasties)	Baopuzi	Ge Hong	Dozens of minerals	Mercury, lead, gold, and sulfur elements	Described distillation, sublimation, and inorganic reactions	①Advanced chemical principles; ②Alchemy pioneered pharmaceutical chemistry	Alchemy flourished; mineral ingestion trend
	Mingyi Bielu	Tao Hongjing	32 mineral medicines were added	-	Established “Jade-Stone” drug category	Systematic mineral classification	Pharmacological system refinement
	Lei gong pao zhi lun	Lei Ji	-	Mica, mercury, and stalactite	First specialized text on mineral drug processing methods	China’s first monograph on concoctions	Maturation of processing techniques
581 CE - 907 CE (Sui and Tang Dynasties)	Newly Revised Materia Medic	Su Jing et al. (22 scholars)	844 drugs (83 minerals)	Red Copper, Green Salt	①First state-compiled pharmacopeia; ②Three-tier mineral classification	National pharmacological standard	Mineral smelting advancements; marine animal bone utilization
	Synonymic Dictionary of Mineral Drugs	Mei Biao	62 chemicals	Xuanhuanghua, Lead Oxide	Compiled Tang alchemical terminologies and synonyms	Definitive guide to Tang alchemy	Codification of alchemical terms
	Essential Formulas for Emergencies	Sun Simiao	104 mineral drugs	Iodine-rich animal thyroids	①Iodine therapy for goiter; ②Mercury ointment for skin diseases	Clinical breakthroughs in mineral medicine	Peak of medical academia
960 CE - 1368 CE (Song Yuanjin Dynasties)	Classified Materia Medica	Tang Shenwei	139 mineral drugs	Vermilion	Expanded clinical use (e.g., arsenic for malaria)	Song pharmacological culmination	Printing boosted medical dissemination; international mineral trade
	Materia Medica Derivations	Kou Zongshi	69 new minerals	-	Detailed mineral properties and functions	Theoretical deepening of pharmacology	Global exchange of mineral medicines
1368 CE - 1912 CE (Ming and Qing dynasties)	Compendium of Materia Medica	Li Shizhen	161 mineral entries	Calamine, gem, diamond	①Comprehensive mineral taxonomy; ②Geographical efficacy correlations	Pioneer of natural classification; integrated 16th-century multidisciplinary knowledge	Decline of Daoist alchemy; rational mineral applications
	Supplements to the Compendium	Zhao Xuemin	38 new minerals	Steel tools	Supplemented Compendium with overlooked minerals	Qing pharmacological expansion	Shift toward pragmatic mineral studies

The 2020 edition of the Pharmacopoeia of China contains a total of 1,607 prescription preparations, among which 376 are herbomineral preparations that include both herbs and minerals, accounting for about 23.40%. In Particular, 31 preparations that are purely mineral-based medicines were recorded in the 2020 Pharmacopoeia (Jacky, 2023). In other words, mineral medicines continuously play central roles in TCMM, each with specific therapeutic uses (Chen et al., 2016).

**FIGURE 1:**
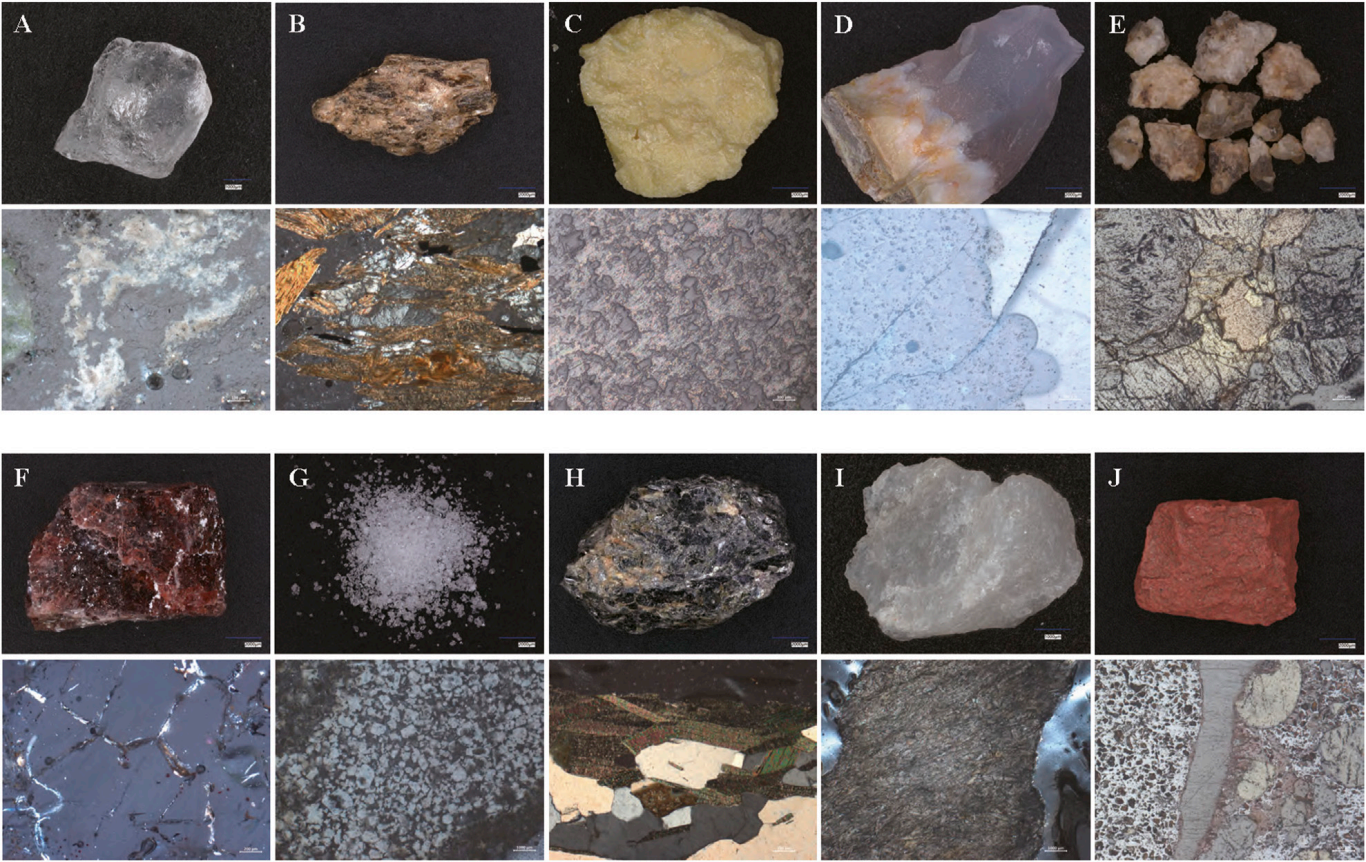
Ten representative Traditional Chinese Mineral Medicines (The stereo microscopy and polarized light microscopy photos of **(A)** Alum; **(B)** Lapis micae aureum; **(C)** Sulfur; **(D)** Agate; **(E)** Maifanstone; **(F)** Sal Ammoniac; **(G)** Borax; **(H)** Lapis Chloriti; **(I)** Actinolite; **(J)** Oreds; The minerals were sourced from the Research Center for Chinese Medicine Processing, Institute of Chinese Materia Medica, China Academy of Chinese Medical Sciences. The authentication of the medicinal materials was conducted by Professor Zhijie Zhang and Assistant Researcher Lu Luo. The polarized light microscopy images were captured using a ZEISS AxioScope.A1 microscope, equipped with a reflected light source and a single-polarized light system).

To systematically evaluate the integration of mineral-based drugs into contemporary healthcare, Table 2 provides a comprehensive analysis of 10 pharmacopoeial mineral medicines, delineating their historical utilization, mechanistic elucidation through modern pharmacology, and the safety and toxicity. Representative examples include *Cinnabaris*, historically employed for sedative and anxiolytic effects; *Gypsum Fibrosum*, utilized for antipyretic and anti-inflammatory properties; and *Realgar*, prescribed for antimicrobial and antiparasitic actions. Figure 1 complements this analysis by presenting macroscopic specimens and polarized light microscopy (PLM) images, which reveal distinct morphologies and crystalline structures essential for quality control and pharmacognostic identification. The specimens analyzed in this study were derived from authenticated samples curated by the Department of Pharmaceutics Processing Research, Institute of Chinese Materia Medica, China Academy of Chinese Medical Sciences, with material authentication performed under the guidance of Prof. Zhang Zhijie (Senior Researcher) and Dr. Luo Lu (Assistant Researcher) following standardized pharmacopoeial protocols. To ensure experimental reproducibility, PLM imaging was systematically conducted using a ZEISS AxioScope.A1 polarizing microscope configured with Köhler illumination, where observations were made under reflected light mode (λ = 550 ± 10 nm) with monochromatic single-polarization settings maintained throughout all analyses.

**TABLE 2:** Representative mineral medicines in traditional Chinese medicine (TCM): Traditional uses, scientific evidence, and safety.

Mineral name	Traditional uses	Scientific evidence	Safety/Toxicity	References
Gypsum	Clears heat, reduces fever, treats headaches, inflammatory conditions	Calcium sulfate dihydrate (CaSO_4_·2H_2_O); anti-inflammatory, antipyretic properties	Safe in controlled doses; excessive use may cause gastrointestinal issues	Chen and Chen (2004), Madeira et al. (2012)
Magnetite	Calms the mind, treats dizziness, tinnitus; strengthens kidney function	Iron oxide (Fe_3_O_4_); magnetic properties linked to grounding effects; potential neuroprotective activity	Non-toxic; contraindicated in patients with iron overload disorders	Hsu, 2001; Marshall (2020)
Dragon Bone	Anchors the spirit, treats anxiety, insomnia, night sweats, chronic diarrhea	Fossilized bone (calcium phosphate); historical use for calcium supplementation	Ethical concerns (fossil sourcing); modern use rare due to conservation policies	Chen and Chen (2004)
Cinnabar	Sedative for anxiety, insomnia; detoxifies	Mercury sulfide (HgS); antimicrobial effects observed *in vitro*	High mercury toxicity; strictly regulated; banned in some formulations	Bensky et al. (2015), Unschuld (2009)
Realgar	Treats parasites, skin infections; antidote for poisons	Arsenic sulfide (As_4_S_4_); antiparasitic and antimicrobial activity	Arsenic toxicity; restricted use; requires detoxification processing	Bensky et al. (2015), Liu et al. (2020)
Alum	External: antiseptic, anti-itching; Internal: diarrhea, epilepsy	Potassium aluminum sulfate (KAl(SO_1_)_2_·12H_2_O); astringent and antimicrobial properties	Overuse may cause aluminum accumulation; regulated in internal use	
Sulfur	Treats scabies, constipation; warms yang	Elemental sulfur (S); antifungal and laxative effects	Toxic in high doses; modern formulations prioritize external application	
Borax	Clears heat, resolves phlegm; treats sore throat, cough	Sodium borate (Na_2_B_4_O_7_·10H_2_O); mild antiseptic and expectorant	Boron toxicity risk; limited to low-dose prescriptions	
Maifan Stone	Detoxifies, promotes tissue regeneration; treats skin disorders, diabetes	Silica-rich igneous rock; trace elements (Fe, Zn) may support metabolic functions	Generally safe; lacks comprehensive toxicity studies	
Actinolite	Treats impotence, joint stiffness, muscle atrophy	Calcium magnesium silicate [Ca_2_Mg_5_(Si_4_O_11_)_2_(OH)_2_]; traditional use for musculoskeletal disorders	Potential asbestos-like fiber risk; rare in modern formulations	

Ongoing research aims to refine the safety of these treatments, understand the difference in materials, and balance traditional knowledge with modern standards (Chen J. et al., 2022). For example, some research explored the differences between grafted Kynam agarwood and regular agarwood to better understand their potential medical applications (Chen et al., 2023). Other studies were being conducted to reduce toxic side effects while preserving the therapeutic properties of minerals like cinnabar and realgar (Guan et al., 2022) and investigated the effects of realgar on gut microbiota to identify the potential biomarker (Luo M. et al., 2023). In summary, due to the development of modern medicine, the use of highly toxic minerals like mercury and arsenic declined due to their well-documented health risks. However, many non-toxic minerals, such as gypsum and magnetite, remain in use today as part of contemporary TCM practices. Contemporary regulations in China and other countries have enforced strict safety guidelines to control the use of minerals, particularly those with heavy metal content (Madeira et al., 2012; Murillo et al., 2022). Mineral medicine continues to be an integral part of TCM, especially in formulations aimed at treating specific conditions related to heat, inflammation, and emotional imbalance (Choy et al., 2008). Additionally, modern research is increasingly investigating the pharmacological properties of these minerals, often validating their traditional uses through biochemical analysis (Shinzhen, 2006; Murillo et al., 2022).

## 3 The application and research on metallodrugs

### 3.1 The history and development of metallodrugs

Twelve metals are essential for humans (Ca, Mg, K, Na, Fe, Zn, Cu, Mn, Mo, Co, Se, Cr) (Da Silva and Williams, 2001)., and human body has developed diverse transportation and metabolic pathways for these essential metals. Although this diversity amounts to a core challenge for the systematic development of metallodrugs, it also highlights the potential of metal and metallodrugs in treating diseases (Haraguchi, 2017; Mjos and Orvig, 2014; Meiling et al., 2013). To date, only several metallodrugs have developed by pharmaceutical industry. At first glance, these metallodrugs seem to represent only a small fraction of all pharmaceuticals; however, some of them are among the most used and important drugs in modern medicine (Clarke et al., 1999). Significantly, this pharmacological lineage traces back to ancient systems: Ayurvedic Rasashastra texts documented mercury detoxification through sequential calcination (puta), while Traditional Chinese Mineral Medicine (TCMM) employed arsenic sulfides (e.g., realgar) for inflammatory conditions—both anticipating modern strategies for toxicity mitigation. Especially, some medical conditions are only treatable with metal-based drugs, which will be discussed in the following chapters (Rosenberg et al., 1965; Kean and Kean, 2008; Hartinger and Dyson, 2009). Notably, the empirical foundations of metal processing in traditional medicine paralleled contemporary biochemical insights (Clifford et al., 2016). Despite its unquestionable success in medicine and historically proven use of metals in the pharmaceutical field, which traces back to the ancient civilizations of Mesopotamia, Egypt, India, and China (Meiling et al., 2013), metallodrugs are less developed compared to small organic molecules in traditional medicinal chemistry or biological molecules. Medicinal inorganic chemistry is underestimated or barely known by many chemists. Metals are still equivocally seen as only toxic agents with no application in medicine and drug development by pharmaceutical industries relies almost entirely on organic and biological compounds (Hambley, 2007; Miranda, 2022).

Metallodrugs are essential to treat a wide range of diseases, of which most have no better alternative treatment. In the late 1800s and early 1900s, the first metal-based drugs were being tested and prescribed to treat many conditions, and many were developed later (Miranda, 2022). This era echoed earlier traditions: Ayurveda’s gold nanoparticles (Swarna Bhasma) utilized citric acid for colloidal stabilization—an ancient precedent for modern gold drug bioavailability enhancement (Pattabhiramaiah et al., 2020). In 1912, Vianna introduced antimony compounds for treating the parasitic disease leishmaniasis (Farrell, 2002). Mercurous chloride (Calomel, Hg2Cl2) has been a well-known diureticum since the Renaissance and was used until the 1950s (Farrell, 2002). Around the same time, P. Ehrlich’s arsenic compounds (As_2_O_3_) were the first successful pharmaceuticals for treating syphilis and gold cyanide (AuCN) was used as a drug against tuberculosis.

Yet, it was only after the clinical approval of cisplatin, in the 1970s, that medicinal inorganic chemistry flourished as a separate field inside inorganic chemistry (Peña et al., 2022). This platinum-based drug, developed in the 1960s and known for its antiproliferative properties, remains the first choice for many cancer treatments (Jurkovicova et al., 2022; Rosenberg et al., 1969; National Cancer Institute, 2007; Brown et al., 2019). Contemporary advances often converge with traditional knowledge: Ayurvedic Tamra Bhasma (nanoparticulate copper) enhances copper bioavailability through samskara processing—a principle now applied in copper-based neurotherapeutics (Usmani et al., 2019). Another example is gold therapies that are applied even today in the treatment of rheumatoid arthritis, both as injections of gold thiolates and orally as auranofin (Hansen and Farver, 2010). Gold (Au) compounds are also used in anti-rheumatoid arthritis (Alessio, 2017). O’Halloran and co-workers show the anticancer activity of small-molecule and nanoparticle forms of arsenic. The recent interest in this class of agents has been fueled by the discovery that diluting the aqueous extraction of arsenic trioxide is now part of the frontline treatment of acute promyelocytic leukemia (Nature Medicine, 2012). This mirrors TCMM’s historical use of arsenic-sulfur compounds (e.g., Xionghuang) in controlled synergistic formulations to mitigate arsenic toxicity (Tian et al., 2019). Over 100 clinical trials involving inorganic arsenic or organoarsenic compounds are currently open, and new generations of both inorganic and organometallic arsenic compounds are under development. To summarize, inorganic compounds including metalloids are a rapidly growing class of agents for the treatment of disease.

The interest in using and application of inorganic chemistry in medicine continues to expand. Since the end of the 20th century, Europe, the United States, and Japan have successively formulated the “Metals in Medicine” program, inorganic drugs and their related basic research have set off a new wave of development (COST D20). At about the same time, a similar program was launched in Japan, with a special issue of Chemical Reviews on pharmaceutical inorganic chemistry published in 1999. A Metal in Medicine program was also launched by the NIH in 2000 (National Institutes of Health, 2000). Major international conferences such as the International Conference on Bioinorganic Chemistry (ICBIC) and the European Conference on Bioinorganic Chemistry (EUROBIC) now contain a significant fraction of presentations dedicated to “metals in medicine” (Coleman et al., 2020). The first Gordon Research Conference dedicated solely to aspects of metals in medicine, an offshoot of the popular Metals in Biology meeting (http://www.grc.org) took place in July 2002 (Meiling et al., 2013). Recent dedicated volumes or sections in Metal Ions in Biological Systems, Chemical Reviews, and Coordination Chemistry Reviews further testify to the growing importance of this subject. Metal-based drugs and imaging agents where the central metal ion is usually the key feature of the mechanism of action. This latter class may also be conveniently expanded to include those radionuclides used in radio-immunoimaging and radioimmunotherapy (Farrell, 2002; Crans and Meade, 2013).

### 3.2 Metabolism and transformation of metallodrugs in the body

The efficacy and toxicity of metallodrugs are not only determined by their chemical structure and activity *in vitro* but are also influenced by their absorption, distribution, metabolism, and excretion (ADME) processes in the body (Dollwet et al., 1985). Due to their unique metal ion composition, metallodrugs have more complex mechanisms of metabolism and transformation in the body compared to traditional small molecule drugs, which is critical for their clinical application. Understanding the metabolic pathways of metallodrugs helps not only to reveal their mechanisms of action but also provides a theoretical foundation for the design and improvement of new drugs (Eckardt et al., 2009).

#### 3.2.1 Absorption

The absorption of metallodrugs involves their transport and bioavailability within the body, typically through oral, injection, or topical administration (Friedman et al., 1992). For orally administered metallodrugs, such as platinum-based drugs, they are primarily absorbed through the gastrointestinal epithelial cells and may form complexes with proteins, enzymes, or other metal ions in the gastrointestinal tract (Binks, 1988). These complexes help enhance the stability and bioavailability of the drugs (Ge et al., 2011). Metallodrugs administered by injection directly enter the bloodstream and are rapidly distributed to various organs, especially tumor tissues. In plasma, metallodrugs often bind to carriers such as transferrin, aiding in their stabilization and targeting of specific regions (Gelot et al., 2023). Metal ions enter cells via specific transport proteins, such as copper transporter CTR1, or may be taken up through endocytosis, especially when metal nanoparticles or nano-drugs are used. These drugs can penetrate the cell membrane’s lipid bilayer (Clifford et al., 2016). Therefore, the absorption mechanisms of metallodrugs are influenced by factors such as their chemical form, molecular size, and hydrophilicity or hydrophobicity (Hambley, 2007).

#### 3.2.2 Distribution

The distribution of metallodrugs refers to the process by which they are distributed in various tissues and organs after entering the body (Haraguchi, 2017). The distribution of these drugs is influenced by multiple factors, including their chemical form, molecular weight, hydrophilicity and hydrophobicity, blood flow, and the barrier properties of target organs. For example, platinum-based drugs such as cisplatin, after binding to plasma proteins like albumin and transferrin, accumulate at high concentrations in organs such as the liver, kidneys, and tumor tissues, where they have a higher affinity (Messori and Merlino, 2016). The distribution of metallodrugs is also affected by the blood-brain barrier and the placental barrier, making it difficult for some drugs to cross these barriers (Huang N. et al., 2024). In addition to traditional distribution methods, metal nanoparticles or nanodrugs can accumulate in specific tissues, such as tumors, through the enhanced permeability and retention (EPR) effect, improving targeting efficacy. The transport of metal ions is also regulated by transport proteins on the cell membrane (Jumper et al., 2021); for example, copper transporter CTR1 aids in the uptake of copper ions and copper-containing drugs, while transferrin helps distribute iron-based drugs (Clifford et al., 2016). Overall, the distribution of metallodrugs depends on their chemical properties, route of administration, and the collaborative action of specific transport systems (Karati et al., 2024).

#### 3.2.3 Metabolism

##### 3.2.3.1 Metal redox reactions

The metabolism of metallodrugs often involves redox reactions, especially in the reduction or oxidation of metal ions within the body (Kean and Kean, 2008). Metals such as iron and copper possess rich redox properties, enabling them to participate in various biochemical reactions in the body (Kohn, 2004). For example, during metabolism, iron can generate free radicals through the Fenton reaction, which can induce oxidative stress within cells, leading to cellular damage (Jomova and Valko, 2011). The metal center of the drug often undergoes changes in its oxidation state (e.g., from Fe(III) to Fe(II)), allowing it to interact with other molecules (Nallappan et al., 2001). This redox process not only influences the biological activity of the drug but also regulates its efficacy and toxicity *in vivo* (Li L. et al., 2021).

##### 3.2.3.2 Dissociation and transformation of coordination compounds

Another key pathway in the metabolism of metallodrugs is the dissociation and transformation of coordination compounds. Inside the body, metal ions typically form coordination complexes with ligands, and these complexes can dissociate or undergo transformation (Lu et al., 2022). For instance, platinum-based drugs like cisplatin, once inside the body, may undergo coordination reactions with plasma proteins, DNA, or other macromolecules to form stable metal-ligand complexes. Under specific conditions, these complexes can dissociate, releasing free metal ions or ligands that then enter different metabolic pathways (Clarke et al., 1999). This process is crucial for the drug’s biological effects, as the release of metal ions may promote binding with biological targets, thereby enhancing the therapeutic effect (Luo L. et al., 2023).

##### 3.2.3.3 Interaction of metal ions with enzymes

The interaction of metal ions with enzymes is another important mechanism in the metabolism of metallodrugs (Manzotti et al., 2000). Many metallodrugs exert their biological activity through binding with enzymes. Metal ions can interact with the active centers of enzymes, modulating their catalytic activity or even inhibiting enzyme function (Petanidis et al., 2019). For example, copper and zinc ions are essential components of many enzymes, playing roles in various metabolic reactions in the body (Mejía et al., 2018). During the metabolism of certain metallodrugs, the metal ions in the drugs may compete for binding to the metal centers of enzymes or interfere with their normal functions, which can significantly impact the drug’s efficacy and side effects (Clifford et al., 2016). In the case of metal-based anticancer drugs, for example, metallodrugs may interact with DNA repair enzymes or detoxifying enzymes, potentially inhibiting the repair mechanisms in tumor cells, thereby enhancing their anticancer effects (Mejía et al., 2018).

#### 3.2.4 Excretion

The excretion of metallodrugs primarily occurs through the kidneys, bile, and other excretion pathways (Misset et al., 2000). The kidneys are the main organs for the excretion of metallodrugs, especially after metal ions and their coordination compounds have been metabolized in the body (Rosenberg et al., 1965). These compounds are often excreted in the urine in the form of free metal ions or water-soluble complexes (Mjos and Orvig, 2014). The reabsorption and secretion of metal ions in the renal tubules influence the rate of excretion (O'Dowd et al., 2024). Certain metallodrugs, such as platinum-based drugs, can bind with molecules in the urine to form larger complexes, which may slow down their excretion, increasing the burden on the kidneys and potentially leading to renal toxicity (Boulikas et al., 2007). Additionally, some metallodrugs are excreted through bile into the digestive tract, with certain metabolites and complexes being eliminated from the body via this route. The excretion mechanisms of metallodrugs are crucial in clinical applications, as they directly affect the drug’s clearance rate and potential toxicity, particularly during long-term or high-dose treatments (Ge et al., 2011). Special attention must be paid to the functional state of the kidneys and other excretory organs.

#### 3.2.5 Effects of in vivo metallodrug transformation

The metabolism and transformation of metallodrugs in the body not only influence their therapeutic efficacy but also their potential toxicity (Sava et al., 2002). For instance, platinum-based drugs like cisplatin may generate hydrophilic metabolites during metabolism, which can lead to kidney damage (Pattabhiramaiah et al., 2020). On the other hand, metal nanoparticles, upon metabolic transformation, may release metal ions that exhibit stronger cytotoxicity. Therefore, it is crucial to thoroughly study the metabolites of metallodrugs and their potential biological effects, as these transformations can significantly impact both the therapeutic outcomes and side effects of the drugs (Schuh et al., 2012).

### 3.3 Mechanism of metallodrugs

**FIGURE 2:**
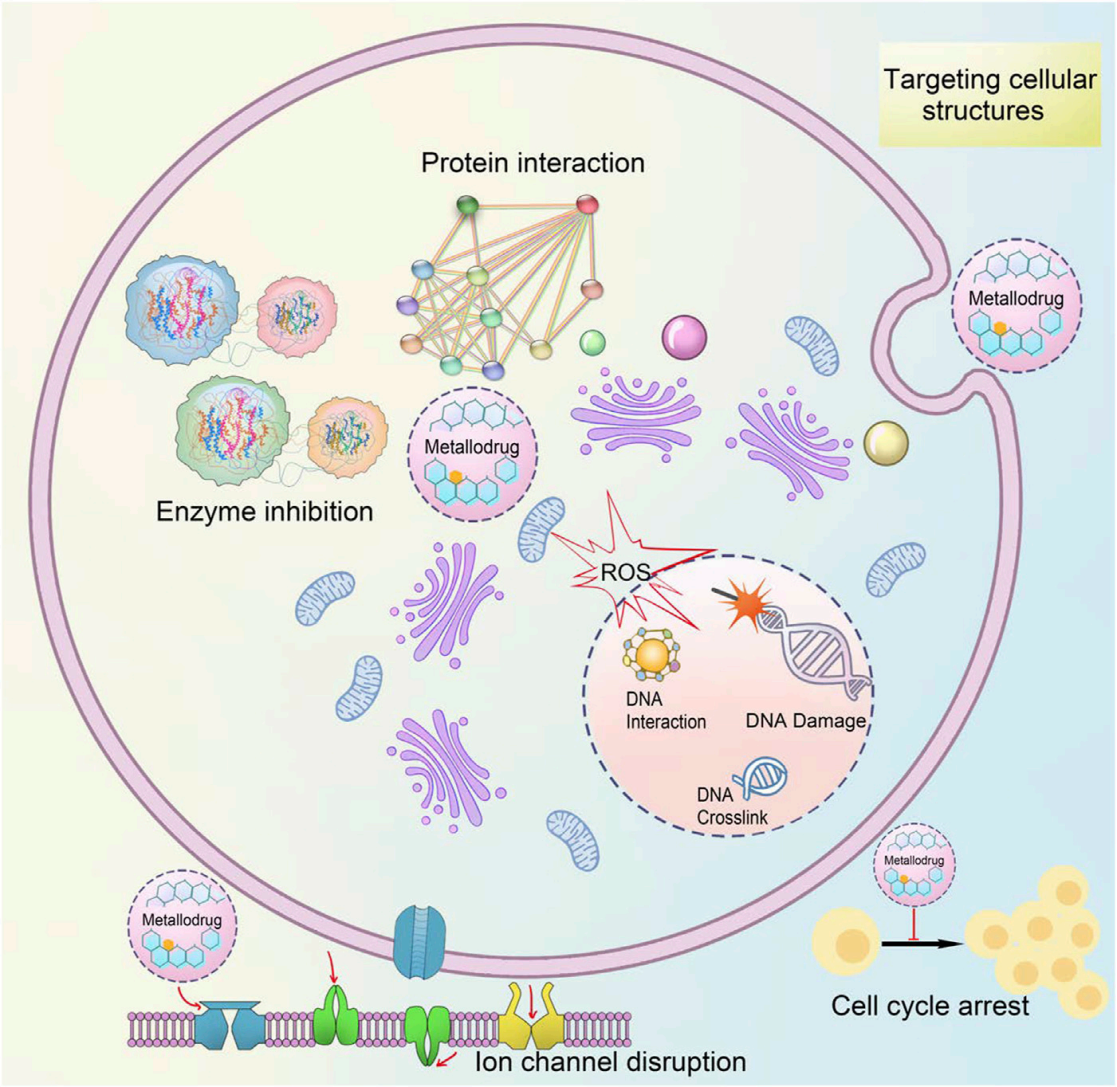
The mechanism and targets of metallodrugs.

Metallodrugs play crucial roles in various biomedical applications, including cancer treatment, antimicrobial activity, and diagnostic imaging (Sheldon, 2017). Their mechanisms of action are diverse and often related to the specific properties of the metal center, such as redox potential, coordination geometry, and the ability to form reactive species. Metallodrugs operate via multiple mechanisms, including protein interaction, enzyme inhibition, DNA interaction, ROS generation, and targeting cellular structures like mitochondria and membranes (Figure 2) (Mejía et al., 2018; Xiong et al., 2022). To summarize, many metallodrugs have more than one target molecule or one mechanism of action. Multiple target molecules and multiple actions give inorganic drugs a combined pharmacological effect (Shizhen, 2006).

Figure 2. The mechanism and targets of metallodrugs.

Schematic representation of the mechanism of metallodrugs affecting target cells. Including enzyme inhibition, protein interaction, ion channels, cell cycle arrest, and ROS (Stathopoulos et al., 2005).

Also, metallodrugs can be classified based on the metal involved, such as platinum, gold, ruthenium, silver, and titanium-based drugs (Table 3), each exhibiting distinct therapeutic effects in cancer, antimicrobial treatments, or diagnostics, which will not be discussed in this review.

**TABLE 3:** Representative metallodrugs in different categories.

	Metal	Name of the drug/trade name	Chemical component	Chemical formula	Indication	Mechanism	
Antitumor drugs	Platin	Platinol, Platinol-AQ	Cisplatin; cis-diamminedichloroplatinum (II) (CDDP)	PtCl2(NH3)2	Testicular cancer: Ovarian cancer; Bladder cancer, lung; Head and neck cancer	Inhibition of DNA replication; Apoptosis; High mitotic index	Frezza et al. (2010)
		Paraplatin	Carboplatin	C6H12N2O4Pt	Ovarian cancer: Lung cancer; Head and neck cancer	Similar to cisplatin, carboplatin causes DNA damage, but with reduced side effects. It forms DNA adducts that inhibit transcription and cell division, triggering apoptosis	Peña et al. (2022)
		Eloxatin	Oxaliplatin	C8H14N2O4Pt	Colorectal cancer	Inhibition of DNA replication; Apoptosis; High mitotic index	Petanidis et al. (2019)
		Nedaplatin/Aqupla	diammine-glycolatoplatinum compound	C2H8N2O3Pt	head, neck, testicular, lung, esophageal, ovarian and cervical cancers	Inhibition of DNA replication; Apoptosis; High mitotic index	Shimada et al. (2013)
		Ormaplatin	tetraplatin, codenamed NSC 363812	C6H14Cl4N2Pt+2	Cisplatin resistant cancers	Inhibition of DNA replication; Apoptosis; High mitotic index	Xia et al. (2025)
		Iproplatin	dichloro-dihydroxy-bis (isopropylamine) platinum (IV)	C6H20Cl2N2O2Pt-4	Trials discontinued	Inhibition of DNA replication; Apoptosis; High mitotic index	Volckova et al. (2008)
		Triplatin tetranitrate/BBR 3464	BBR 3,464 is a charged (+4), triplatinum complex whose structure derives from that of trans-diammindichloroplatinum (II), in which the bridges between the Pt (II) ions are represented by 1,6-diaminohexane	C12H50Cl2N14O12Pt3	Trials discontinued. NSCLC, Ovarian cancer	Inhibition of DNA replication; Apoptosis; High mitotic index	Allardycec and Dyson (2016)
		Phenanthriplatin	cis-[Pt (NH3)2-(phenanthridine)Cl]NO3	C13H15ClN4O3Pt	Solid tumors	Inhibition of DNA replication; Apoptosis; High mitotic index	O'Dowd et al. (2024)
		Picoplatin	azane; 2-methylpyridine; platinum (2+); dichloride	C6H10Cl2N2Pt	Metastatic colorectal cancer	Inhibition of DNA replication; Apoptosis; High mitotic index	Bingham and Cohrssen (2012), Rahman and Singh (2019)
		Satraplatin	(OC-6–43)-bis(acetato)amminedichlorocyclohexylamine platinum (IV)	C10H22Cl2N2O4Pt	Breast cancer; Lung cancer; Prostate cancer; Radiotherapy	Inhibition of DNA replication; Apoptosis; High mitotic index	Doshi et al. (2012)
		Heptaplatin/Sunpla	malonate as a chelating leaving group and of 2-(1-methylethyl)-1,3-dioxolane-4, 5-dimethanamine as a chelating group	C11H20N2O6Pt	Collorectal cancer	Inhibition of DNA replication; Apoptosis; High mitotic index	Huang et al. (2021)
		Lobaplatin	1,2-diammino-l-methyl-cyclobutane-platinum (II)-lactate	C9H18N2O3Pt	Hepatocellular cancer	Inhibition of DNA replication; Apoptosis; High mitotic index	Wu et al. (2010)
		Nanoplatin/NC-6004	Cisplatin micellar nanoparticle	-	NSCLC; Biliary tract cancer; Bladder cancer	Inhibition of DNA replication; Apoptosis; High mitotic index	Volovat et al. (2020)
	Copper	Elesclomol	N-malonil-bis(N-metil-N-tiobenzoyl hidrazide)	C19H20N4O2S2	Refractory solid tumors; Ewing sarcoma	Oxidative stress; Apoptosis; Cell redox system	Bruijnincx and Sadler (2008)
		Casiopeina III-ia	Cu complexes; [Cu(N-N) (X-Y)H2O]NO3, where N-N is a diimine ligand (phenanthroline or dipyridyl) and X-Y is a bidentate ligand (acetylacetone, salicylaldehyde, peptide, benzimidazole)	[Cu(N-N) (X-Y)H2O]NO3	AML; Colon cancer; Cervical cancer	Oxidative stress; Apoptosis; Cell redox system	Aguilar-Jiménez et al. (2022)
	Ruthenium	NAMI-A	[ImH][trans-RuCl4(DMSO) (Im)] (Im = imidazol, DMSO = dimetilsulfoxid)	C8H15Cl4N4ORuS	Trials discontinued	RAPTA-T; Antimetastatic activity; Apoptosis	Alessio (2017)
		KP1019	[InH][trans-RuCl4(In)2] (In = indazol)	C21H19Cl4N6Ru	Breast cancer; Colorectal cancer	RAPTA-T; Antimetastatic activity; Apoptosis	Hartinger et al. (2008)
		Ru(II)-diphosphine complexes containing Lapachol (Lap) and Lawsone (Law)	[Ru(Lap) (dppm)2]PF6; [Ru(Law) (dppm)2]PF6	-	New potential anticancer agents	RAPTA-T; Antimetastatic activity; Apoptosis	Kabir et al. (2023)
	Vanadium	Experimental	Vanadium complexes; [V(HCys)3]	[V(N-N) (maltol)2]ClO4; [(VCl(Phen)2)2O]2+; [(VCl(Bpy)2)2O]2+ (Bpy/Phen = bipyridine/o-phenanthroline)	New potential anticancer agents	[Apoptosis; Low therapeutic index; ROS; Haber-Weiss chemistry; V10O286−, (decavanadate)	Kumar et al. (2024)
	Radium	Xofigo	Alpharadin	223RaCl2	Skeletal metastases	Alpha particles destroys cancer cells	Parker et al. (2018)
	Titanium	Salan based Titatnium complexes	diamino bis-phenolato titanium (IV) complexes	-	Tumors	The drug binds to DNA, causing cell cycle arrest and apoptosis in cancer cells. Exhibits lower toxicity than platinum-based drugs and has shown some promise in drug-resistant tumors Afffinity to DNA; Apoptosis; Drug-resistant tumors	Zhao et al. (2023)
	Technetium	PoltechDTPA	DTPA (diethylenetriaminepentacetate)	C14H23N3O10	Contamination with plutonium, americium and curium; MRI contrast	Radiopharmaceutical drug	Kanal (2016)
	Gallium	Experimental	Gallium complexes	[tris(3-hydroxy-2methyl-pyrone)]	Iron dependet cancers	Binds to transferrin; Less toxic than platinum	Chitambar (2018)
	Arsenic	Pishuang	Arsenic trioxide	As2O3	Approved for treating APL, a subtype of leukemia	Apoptosis; Oxidative stress; degradation of PML-RARα	Jiang et al. (2023)
	Gold	Experimental	Amino-linked heterocyclic carbene gold (Au) complex	-	Chemoresistant tumors	ROS; Inhibition of TrxR	Kabir et al. (2023)
	Zinc	ZINC000013513540-JAK2 and ZINC000004099068-JAK2	Zinc complexes	-	Primary myelofibrosis (PMF)	Act the same as Fedratynib; JAK2 inhibitor	Li W. et al. (2021)
Antidiabetic drugs	Zinc	Zincate, Orazinc, Solvazinc	Zinc Sulfate, Zinc Gluconate, Cystotine, Metformin	ZnSO4; C12H22O14Zn,; C4H5N3O; C4H11N5	Diabetes	PI3K; Akt; GLUT4; Inhibition of gluconeogenesis	Gabriel et al. (2021)
	Vanadium	Vanadyl Sulfate, “vanadium” or “vanadyl sulfate”, Vanadyl Complex” Sodium metavanadate	Vanadyl sulfate and sodium metavanadate	H10O10SV; NaVO3	Diabetes	Mimics insulin; IR; PTPs; GLUT4; PEPCK	Amaral et al. (2023)
	Chromium	Chromax “Chromium Picolinate” (generic) “Cr-Picolinate” “GTF Chromium”	Chromium Picolinate	C16H12CrN3O6	Diabetes	Improve insulin sensivity; GLUT4; Lower serum glucose	(Peng and Yang, 2015; Hua et al., 2012))
	Copper	Copper (II) complexes	Copper Sulfate/complexes	CuSO4	Diabetes	Scavenging free radicals; Modulation of insulin signaling	Omoregie et al. (2022)
	Molybdenum	Molybdenum Supplements	Two molybdenum complexes, MoHL1 and MoHL2, were synthesized using tetradentate salen-type (ONNO) ligands HL1 and HL2 as precursors. These ligands (HL1 and HL2) were derived from the condensation of 3,5-dichlorosalicylaldehyde with substituted o-phenylenediamines	MoHL1; MoHL2 (HL1 and HL2 represent tetradentate salen-type (ONNO) ligands.)	Diabetes	Purine degradation; Metabolism of sulfur-containing amino acids; Carbohydrate metabolism; Oxidative stress	Saravanan and Sheela (2024)
	Cobalt	Cobalt (III) complexes; Cobalt Chloride	cobalt-quercetin complex’s (CQC)	CoCl2	Diabetes	GLUT1; GLUT4; Insulin-mimetic action; HIF; Glycolysis	Hassanien et al. (2020)
Antiparasitic drugs	Antimony	Pentostam, Glucantime	Sodium Stibogluconate and Meglumine Antimoniate; NEW EXPERIMENTA DRUG: Sb2O5·nH2O NPs	Sb2O5·nH2O	leishmaniasis	Inhibits trypanothione reductase; ROS; Glycolysis; Apoptosis	Franco et al. (2016)
	Platinum	Experimental	Pt and Pd organometallic hit compounds	[MII(dppf) (mpo)](PF6), where M = Pt or Pd	Trypanosoma brucei, Leishmania	Inhibition of DNA replication; A parasite enzyme absent in the host, NADH-fumarate reductase, had been *in vitro* identified as a potential target for treatment with [MII(dppf) (mpo)](PF6), where M = Pt or Pd	Scalese et al. (2022)
	Ruthenium	Experimental	trithiolato bridged dinuclear Ru(II) arene conjugated with 9–(2–oxyethyl)–adenine unit; other ruthenium complexes	[(η6-p-MeC6H4Pri)2Ru2 (µ2-SC6H4-p-CH3)3]Cl, [(η6-p-MeC6H4Pri)2Ru2 (µ2-SC6H4-p-But)3]Cl and [(η6-p-MeC6H4Pri)2Ru2 (µ2-SCH2C6H4-p-But)2-(µ2-SC6H4-p-OH)]BF4	Plasmodium and Trypanosoma	Interefering with iron-dependent processes; ROS	Chakraborty et al. (2024)
	Artemisinin	Artemisin; however there are new derivates: OZ439, OZ277, NITD609	Ferrocene-Artemisinin Conjugates, Gold-Artemisinin Complexes	C15H18O4	Plasmodium falciparum	Alkylate heme as target	Mäser et al. (2012)
	Gold	Auranofin	Ridaura	C20H34AuO9PS0	Amoebiasis (caused by Entamoeba histolytica); Giardiasis: HIV infection, COVID-19; cancers	Inhibitor of USP14 and UCHL5 (USP14/UCHL5)	Feng et al. (2020)
	Iron	Nitrofurtimox	Ferrocene-Artemisinin Conjugates (experimental stage)	C10H13N3O5S	antiplasmodial, anti Chagas and antitripanosoma	Source of iron for Artemisin; ROS	Ludwig et al. (2019)
	Copper	Experimental	Copper (II) complexes (investigational compounds)	[Ag(BZN)2]NO3·H2O (1), [CuCl2(BZN) (H2O)]·1/2CH3CN (2), [Ag(PPh3)2(BZN)2]NO3·H2O (3), and [Cu(PPh3)2(BNZ)2]NO3·2H2O	Leishmania donovani, Trypansoma cruzi, Chagas disease	Little KDNA; Increased autophagy; [44]; [45]	de Souza et al. (2023)
	Silver	Experimental	Silver complexes	Ag(BZN)2]NO3·H2O (1), [CuCl2(BZN) (H2O)]·1/2CH3CN (2), [Ag(PPh3)2(BZN)2]NO3·H2O (3), and [Cu(PPh3)2(BNZ)2]NO3·2H2O	Plasmodium, Leishmania, and Trypanosoma species	Little KDNA; Increased autophagy in nucleus (not frequently reported in the literature)	Luo M. et al. (2023)
	Vanadium	Experimental	Oxidovanadium (IV) compounds	[VIVO(L-2H) (NN)]; [VIVO(BrSalsem) (aminophen)]	Trypanosoma brucei, Leishmania	Intercalates parasite’s DNA	Scalese et al. (2022)
	Pallad	Experimental	Pt and Pd organometallic hit compounds	[MII(dppf) (mpo)](PF6), where M = Pt or Pd	Trypanosoma brucei, Leishmania; A parasite enzyme absent in the host, NADH-fumarate reductase, had been *in vitro* identified as a potential target for treatment with [MII(dppf) (mpo)](PF6), where M = Pt or Pd	Intercalates parasite’s DNA	Scalese et al. (2022)
Antibacterial drugs	Silver	Silvadene	Silver sulfadiazine; silver nanoparticles	C10H9AgN4O2S	Broad spectrum	Increases membrane’s permeability; Interacts with DNA	Nallapan et al. (2001)
		Experimental	Silver nanoparticles	-	Broad spectrum (including multi resisitant bacterias)	Increases membrane’s permeability; Interacts with DNA; ROS;	Franci et al. (2015)
	Gold	Experimental	Gold nanoparticles (AuNPs)	-	Antibiotic-resistant strains	Increases membrane’s permeability; ROS	Gu et al. (2021)
	Copper	Experimental; copper sulfate	Copper sulfate AND copper clusters (CuCs)	CuSO4; CuCs	multi resistant bacterias	Increases membrane’s permeability; GSH/GSSG; ROS	Yu et al. (2024)
	Bismuth	Pepto-Bismoll; experimental	Bismuth subsalicylate; bismuth complexes	C7H5BiO4	Primarily used in the treatment of *H. pylori* infections, often in combination with antibiotics	Increases membrane’s permeability; Chelation of bacterial proteins	Wang et al. (2020)
	Cobalt	Experimental	Cobalt nanoparticles (Co. NPs)	-	Broad spectrum (including multi resisitant bacterias)	interaction with thiol groups (-SH); Increased membrane’s permeability; ROS	Abass et al. (2021)
	Zinc	Experimental	Zinc nanoparticles conjugated with common and popular antibiotic drugs (for instance Ceftriaxone)	-	multi resistant bacterias	Conjugated with Ceftriaxone and Ampicilin	Akbar et al. (2021)
	Gallium	Experimental	Gallium nitrate; gallium complexes and vesicles containing gallium	Ga(OH)3; Ga(OH)4	under the research	Mimics iron; disruption of bacterial enzymes	Li et al. (2022)
	Iron	Experimental	Iron oxide nanoparticles	(Fe_3_O_4_-NPs)	Targeting biofilm-forming bacteria and showing potential in combating multidrug-resistant bacteria	Increases membrane’s permeability; ROS	Gudkov et al. (2021)
	Ruthetium	Experimental	Ruthetium complexes (for instance ruthenium polypyridyl)	C30H24N6Cl2Ru·6H2O	under the research (potentially suitable for MRSA)	Intercalation to DNA and enzymes; ROS	(Chen Y. et al., 2022; Bu et al., 2020)

#### 3.3.1 Protein interaction and enzyme inhibition

Proper three-dimensional structure is critical for a protein to perform its biological role. Research indicates that protein misfolding can alter functions and contribute to various diseases (Nevone et al., 2020; Louros et al., 2023; Huang et al., 2023; Huang Y. et al., 2024). One mechanism of metallodrugs is to interact with proteins, especially enzymes. For instance, gold-based drugs like *auranofin* show efficacy in treating rheumatoid arthritis and are being studied for their anticancer properties due to their enzyme-inhibitory mechanisms (Alessio, 2017). *auranofin* is known to inhibit thioredoxin reductase, an enzyme involved in maintaining cellular redox balance (Tan et al., 2020). By disrupting this balance, *auranofin,* and similar metallodrugs induce oxidative stress, which plays a significant role in their anticancer and anti-inflammatory effects (Schuh et al., 2012; Madeira et al., 2012; Kean and Kean, 2008; James et al., 2015). Another example is *cisplatin*, which can form stable coordination complexes with sulfur-containing amino acids such as cysteine, altering protein function and disrupting essential cellular processes (Peng and Yang, 2015; Bruijnincx and Sadler, 2008). Gallium has been used to treat hypercalcemia, a condition commonly found in individuals with cancer (Chitambar, 2012). Although the exact mechanism of gallium requires further investigation, it is known to interact with transferrin and enter cells, leading to the inhibition of ribonucleotide reductase (de Assis et al., 2022).

Metals have the capability to modulate the structure of some key proteins thus modifying their activity (Tang et al., 2021). A recent study suggests that calcium can mimic estrogen to interact with ligand binding domain of estrogen receptors in breast cancer cells and combine a calcium channel blocker with an antiestrogen reversed resistance to the antiestrogen in breast cancer (Divekar et al., 2011; Cyrus et al., 2021). Therefore, identifying the novel metal binding site can expand the application of metals. For example, TP53 is one of the most frequently mutated genes in cancer, yet these mutations remain therapeutically challenging due to the diverse mechanisms of inactivation and the lack of universally targetable sites (Tian et al., 2019). A recent study suggests that arsenic trioxide can restore the function of mutated p53 through an allosteric site, offering a potential pathway for novel cancer therapies (Chen et al., 2021).

Additionally, metalloproteins have emerged as significant drug targets due to their vital roles in metabolism and genetic information transfer (Usmani et al., 2019). These proteins, which contain metal ions as key components, are involved in a variety of cellular functions. For example, ribonucleotide reductase, a diiron enzyme necessary for DNA synthesis, is a well-known drug target, with efforts focused on disrupting the active site’s iron moieties (Medici et al., 2015). Zinc, the second most prominent trace metal in the human body after iron, plays a critical role in numerous enzymatic processes, fulfilling both structural and catalytic functions. Zinc’s involvement in DNA transcription, hydrolysis, and catalysis has made zinc-containing proteins, such as matrix metalloproteinases (MMPs) and zinc-finger proteins, attractive targets for chemotherapy, particularly in cancer and HIV treatments (Medici et al., 2015). MMPs, enzymes involved in extracellular matrix degradation, play a crucial role in cancer metastasis. Their activity is inhibited by endogenous tissue inhibitors of metalloproteinases (TIMPs). MMP inhibitors, such as batimastat, have been developed to bind competitively to the zinc active sites, inhibiting the abnormal regulation of MMPs and thus potentially preventing metastasis (European Cooperation in Science and Technology, 2012). Similarly, zinc-finger proteins, which bind zinc ions to stabilize their structures, have been targeted with metal-based compounds like aurothiomalate, which disrupt the zinc-cysteine interactions in the protein’s active site, providing a novel approach to chemotherapy and against HIV (Medici et al., 2015).

#### 3.3.2 DNA interaction

Metallodrugs have emerged as powerful tools in therapeutic applications, particularly for cancer treatment and antimicrobial therapies, due to their ability to interact with biological systems through various mechanisms (Wadekar et al., 2005). One of the key mechanisms is the binding of metallodrugs to DNA (Janoš et al., 2021). Platinum-based drugs such as cisplatin, carboplatin, and oxaliplatin are widely employed in oncology for their DNA-binding properties by forming covalent bonds to guanine sites, which prevent DNA replication and cause DNA distortion (Alessio, 2017; Wheate et al., 2010). This disruption prevents essential processes such as DNA replication and transcription, which ultimately leads to apoptosis. Cisplatin and its analogs, including oxaliplatin and carboplatin, are utilized in combination therapies for various cancers, while other compounds like lobaplatin and nedaplatin are predominantly used in Asia (Jurkovicova et al., 2022; Tsvetkova, and Ivanova, 2022; Pignata et al., 2021; Rottenberg et al., 2021). Efforts to develop non-classical platinum compounds, which form different types of platinum-DNA adducts, have led to the discovery of new platinum-based agents with unique clinical activity. For example, a trinuclear platinum agent has been studied in clinical trials (Medici et al., 2015). Titanium-based compounds such as *titanocene dichloride* are being investigated for their reduced toxicity compared to platinum-based drugs and their ability to induce oxidative stress via DNA interaction (Chen Y. et al., 2022). Other metal complexes, such as ruthenium, function through DNA intercalation, where they insert between base pairs without forming covalent bonds, leading to the impairment of DNA function. In addition, certain metal complexes, such as those containing copper or iron, promote the formation of reactive oxygen species (ROS), which also cause oxidative damage to DNA, further inhibiting cellular proliferation and inducing cell death (Johnstone et al., 2016; Simpson et al., 2019).

#### 3.3.3 ROS generation

A further crucial mechanism involves the generation of ROS, which metallodrugs often induce as part of their therapeutic action. For example, iron-based complexes catalyze the Fenton reaction, producing highly reactive hydroxyl radicals (•OH) that damage cellular components. Moreover, some metallodrugs undergo redox cycling, in which metals like copper, iron, or manganese oscillate between oxidation states, continuously generating ROS (Zhao et al., 2023). This oxidative stress damages DNA, proteins, and lipids, leading to cell death (Fenton, 1894; Tang et al., 2021). Ruthenium-based compounds, such as NAMI-A and KP1019, have shown anticancer potential through mechanisms involving DNA interaction and selective ROS generation in cancer cells, while minimizing damage to healthy tissues. They also hold promise for use in photodynamic therapy (Alessio, 2017; Lainé and Passirani, 2012; Roy and Paira, 2024). Research suggests that arsenic trioxide, used in the treatment of acute promyelocytic leukemia, induces apoptosis and generates reactive oxygen species (ROS) as part of its mechanism of action (Murillo et al., 2022; Yedjou et al., 2010). Iron and copper complexes are also being explored in both cancer and antimicrobial treatments due to their roles in redox reactions that disrupt metabolic processes in cancer cells and induce oxidative stress in pathogens (Murillo et al., 2022). Silver-based drugs, particularly in nanoparticle form, exhibit significant antimicrobial effects through mechanisms involving ROS generation and membrane disruption, making them promising candidates in the fight against resistant pathogens (Franci et al., 2015; Pal et al., 2007).

#### 3.3.4 Targeting cellular structures

In addition to targeting DNA and proteins, metallodrugs can affect cellular structures and organelles. Platinum- and gold-based drugs often localize to mitochondria, where they cause mitochondrial membrane potential collapse (Wang and Lippard, 2005). This induces ROS production and triggers the release of pro-apoptotic factors, leading to apoptosis. Metallodrugs also disrupt cell membranes, as is the case with silver nanoparticles, which alter membrane permeability and cause structural damage, a mechanism particularly effective in antimicrobial treatments. This makes silver-based drugs valuable for their broad-spectrum antimicrobial properties, as they can induce oxidative stress and damage bacterial cell membranes (Lemire et al., 2013; Xiu et al., 2012). Additionally, lanthanide-based compounds, such as gadolinium complexes, are primarily used as imaging agents but are increasingly being explored for their therapeutic potential, particularly in cancer treatment (Jin et al., 2022).

#### 3.3.5 Effects on ion channels

Ion channels serve multiple functions including chemical signaling, transepithelial transport, cytoplasmic regulation, intracellular ion concentration, pH, and cell volume. Thus, dysfunction of ion channels can cause severe diseases (Xiong et al., 2022). Metallodrugs can interact with ion channels by the metal’s ability. When metallodrugs bind to channel proteins, the ion channel activities can be modulated, such as Zinc, which can modulate NMDA receptors (Lee et al., 2023). Another common mechanism of metallodrugs regulating ion channel is acting as ion channel blockers, such as platinum-based drugs cisplatin. Cisplatin a well-known chemotherapy drug, can block potassium channels, specifically KCNQ1 channels (Taukulis et al., 2021). By blocking the channels, cisplatin interferes with potassium ion flow, which disrupts normal neuronal signaling. Metallodrugs can also regulate ion channels via interaction with co-factors or regulators. Cobalt compounds have been shown to modulate hypoxia-inducible factor-1 (HIF-1), a key regulator of oxygen homeostasis that also controls the expression of various ion channels. Through this pathway, cobalt can affect calcium channels and potassium channels, altering cellular responses to hypoxia and potentially providing therapeutic benefits in ischemic conditions (Chachami et al., 2004). Metals in metallodrugs often exhibit redox properties, which can influence ion channels sensitive to changes in oxidative states, for instance, vanadium complexes, like vanadyl sulfate, can affect ion channels by inducing oxidative stress. Vanadium’s redox activity can modulate the activity of sodium-potassium ATPase channels, influencing ion gradients across the cell membrane (Xu et al., 2005). This mechanism is of interest in diabetes research, as vanadium compounds mimic insulin by improving glucose uptake through channel modulation (North and Post, 1984). However, there still some metallodrugs that impact the ion channels and ions with unclear mechanisms. Lithium carbonate, a drug known for more than 100 years, is a commonly used drug to treat patients with unipolar and bipolar depression, and for the prophylaxis of bipolar disorders and acute mania. Although the mechanism is still under investigation, studies suggest that lithium can stabilize mood through ions (Yanagita et al., 2007; Czarnywojtek et al., 2020).

#### 3.3.6 Combination treatment

Many useful drugs contain metal-binding sites, which may alter the physiological profile of the original drugs. For example, the cardiac toxicity of adriamycin is mediated through iron chelation (Potuckova et al., 2014). Cellular uptake of copper-chelated thiosemicarbazones is advanced over that of free ligand because of the enhanced lipophilicity of the metal drug combination. The quinolones are a group of synthetic antibacterial agents related to nalidixic acid. Combining with metals may repurpose existing drugs. Superoxide has been implicated as a mediator of disease states such as inflammation, myocardial ischemia-reperfusion injury, cancer, and AIDS. Superoxide dismutase (SOD) enzymes are critical in removing such oxidative damage. Non-steroidal anti-inflammatory drugs (NSAIDs) such as indomethacin inhibit cyclooxygenase and eventually prostaglandin synthesis. Copper and zinc complexes of NSAIDs may exhibit ‘SOD-like’ activity and may be useful in modulating the properties of the parent drugs. However, this requires further investigation. These interactions with drugs add another dimension to the therapeutic potential of metallodrugs (Farrell, 2002).

Also, there is a shift from targeting a single structure to pharmacological design for multiple pathologic segments. The understanding of the *in vivo* metabolism and transformation of metal complexes has enabled us to gradually grasp the laws of metabolism and transformation of metal compounds (Miranda, 2022). Therefore, metallodrugs will continuously play an important role in the medical treatment.

## 4 Challenges and resolutions for developing metallodrugs

Metallodrugs have the potential to emerge as an important class of therapeutics in modern medicine, primarily due to their unique mechanisms of action and diverse applications (Yatoo et al., 2023). Currently, there are multiple studies on metallodrugs. Research into platinum complexes continues to yield promising results for treating resistant tumors (Eckardt et al., 2009; European Medicines Agency, 2008) and trials for repurposing auranofin in cancer treatment underscore the potential for metallodrugs beyond their traditional applications (Mayo Clinic, 2025; Rao et al., 2011). Due to their multiple functions, especially in cancer treatment, more and more metallodrugs are approved by the FDA (Table 4). It shows us that metallodrugs play a more important role in both research and clinical than before. However, more and more challenges are uncovered and discussed below.

**TABLE 4:** FDA approved drugs that contain metals.

Metal	Name of the drug/trade name	Chemical component	Development stage (FDA_approved/ClinTrial)	Indication
Platin	Cisplatin	Cis-diamminedichloroplatinum (II) (CDDP)	FDA approved	Tumors (Go and Adjei, 1999)
	Carboplatin	Carboplatin	FDA approved	Ovarian cancer; Small cell lung cancer (Go and Adjei, 1999)
	Oxaliplatin/Eloxatin		FDA approved	Colorectal cancer; Organic Cation Transporter 1/2; Copper Transporter 1 (Misset et al., 2000)
	Nedaplatin/Aqupla	Diammine-glycolatoplatinum compound	NCT04834206	Head and neck cancers; Female reproductive tract cancers; Lung cancer; Esophageal cancer (Monneret, 2011)
	Ormaplatin	Tetraplatin, codenamed NSC 363812	Various doses, dose patterns, and modes of administration (intravenous and intraperitoneal) were investigated in six Phase I clinical trials; however, no Phase II clinical trials have been planned	Cisplatin-resistant cancers. (Cornelison and Reed, 1993)
	Iproplatin	Dichloro-dihydroxy-bis (isopropylamine) platinum (IV)	Clinical Trial	Trials discontinued; Equal effectiveness to cisplatin (Friedman et al., 1992)
	Triplatin tetranitrate		NCT00014547; NCT00024362	Solid tumors,Trials discontinued. (Shah and Dizon, 2009)
	Phenanthriplatin	Cis-[Pt (NH3)2-(phenanthridine)Cl]NO3		O'Dowd et al. (2024)
	Picoplatin	Azane; 2-methylpyridine; platinum (2+); dichloride	NCT00465725	Metastatic colorectal cancer (Bingham and Cohrssen, 2012; Rahman and Singh, 2019)
	Satraplatin	(OC-6–43)-bis(acetato)amminedichlorocyclohexylamine platinum (IV)	FDA approved	Breast cancer; Lung cancer; Head and neck cancers; Radiotherapy (Choy et al., 2008)
	Heptaplatin/Sunpla	Malonate as a chelating leaving group and of 2-(1-methylethyl)-1,3-dioxolane-4, 5-dimethanamine as a chelating group	FDA approved	Gastric cancer (Xu et al., 2005)
	Lobaplatin	1,2-diammino-l-methyl-cyclobutane-platinum (II)-lactate	NCT03413436	Small cell lung cancer; metastatic breast cancer, Leukemia, Esophageal cancer (McKeage, 2001)
	BBR 3464	BBR 3,464 is a charged (+4), triplatinum complex whose structure derives from that of trans-diammindichloroplatinum (II), in which the bridges between the Pt (II) ions are represented by 1,6-diaminohexane	NCT00014547, NCT00024362	Non-Small Cell Lung Cancer; Ovarian cancer (Manzotti et al., 2000)
	NC-6004/nanoplatin		Clinical Trial	Non-Small Cell Lung Cancer; Biliary tract cancer; Bladder cancer (Volovat et al., 2020)
Copper	Elesclomol	N-malonil-bis(N-metil-N-tiobenzoyl hidrazide)	NCT01280786	Refractory solid tumors; Ewing sarcoma (Marchetto et al., 2020)
	Casiopeina III and Casiopeina II-gly	Structurally, Casiopeinas are mixed Cu complexes with the general formula [Cu(N-N) (X-Y)H2O]NO3, where N-N is a diimine ligand (phenanthroline or dipyridyl) and X-Y is a bidentate ligand (acetylacetone, salicylaldehyde, peptide, benzimidazole). The representatives selected for preclinical and clinical testing are Casiopeina III (CasIII) and Casiopeina II-gly (CasII-gly)		Acute myeloid leukemia, Colon cancer; Cervical cancer (Akhter et al., 2024)
Ruthenium	NAMI-A	[ImH][trans-RuCl4(DMSO) (Im)] (Im = imidazol, DMSO = dimetilsulfoxid)	NCT04843163	Solid tumor, Trials discontinued. (Sava et al., 2002)
	KP1019 and KP1339	[InH][trans-RuCl4(In)2] (In = indazol) and KP1339 it is KP1019 sodium salt		Breast cancers; Colorectal cancers (Hartinger et al., 2008)
Radium	Xofigo	Alpharadin	FDA approved	Skeletal metastases (Coleman et al., 2020)
Technet	DTPA (diethylenetriaminepentacetate)		FDA approved	Contamination with plutonium, americium and curium; MRI contrast (Ahmad et al., 1995)

### 4.1 Toxicity and side effects

One of the main challenges in metallodrug development is the severe toxicity and off-target effects associated with metal-based compounds. For example, metals such as calcium and cadmium can target receptors, disrupt downstream signaling pathways, and potentially, lead to cancer (Psaltis et al., 2024; Sharawi et al., 2023). Cisplatin is well-known for its nephrotoxicity, neurotoxicity, and ototoxicity, which limit its therapeutic index (Miller et al., 2010). This toxicity primarily stems from the non-specific nature of its mechanism of action, where it binds to DNA in both tumor cells and normal cells, leading to extensive collateral damage.

In developing new inorganic drugs, researchers are addressing the issue of toxicity through various strategies. For example, arsenic compounds, typically considered toxic, can be rendered harmless in certain chemical forms, such as non-toxic methylated arsenic species found in seafood (Medici et al., 2015). Similarly, selenocysteine can reduce oxidative stress toxicity through specific coordination forms (Krakowiak and Pietrasik, 2023). This points to the possibility of designing safer inorganic drugs by controlling their chemical forms and interactions within the body. Additionally, research into gold and copper compounds has shown promising results in treating inflammatory conditions and viral infections, with findings that their metabolites (e.g., gold-thiol complexes) may exert targeted therapeutic effects by modulating thioredoxin reductase activity (Medici et al., 2015; Lu et al., 2022).

One approach to overcome off-target effects is designing metal-based drugs with higher selectivity for tumor cells or disease-affected tissues. Targeted drug delivery systems such as nanoparticle carriers or ligand-targeting systems can enhance the therapeutic index (Farinha et al., 2022). For example, gold nanoparticles conjugated with tumor-specific antibodies (such as anti-EGFR antibodies) have been developed to reduce damage to normal tissues via an active targeting mechanism (Chen et al., 2016). Furthermore, the development of prodrugs—metallodrugs that become active only in the presence of specific stimuli in the tumor microenvironment—holds great promise for reducing systemic toxicity. Recent studies have shown that ruthenium-based prodrugs can be selectively activated under hypoxic conditions, demonstrating over three times the tumor-targeting efficiency of traditional platinum-based drugs (Karati et al., 2024).

### 4.2 Drug resistance

Resistance to metallodrugs, such as cisplatin, remains a significant hurdle in cancer therapy. Tumor cells can develop resistance through various mechanisms, including increased drug efflux, enhanced DNA repair, and altered drug detoxification processes, all of which reduce the efficacy of treatment (Luo et al., 2024). The emergence of multidrug resistance (MDR) also compromises the long-term effectiveness of metallodrugs. Addressing drug resistance requires the combination of metallodrugs with other therapeutic agents, including inhibitors of resistance pathways. For instance, such as the PARP inhibitor olaparib, to significantly enhance the efficacy against BRCA-mutant ovarian cancer (Lord and Ashworth, 2017). Notably, novel metallodrugs, such as ruthenium and titanium complexes, overcome resistance through unique mechanisms of action: the ruthenium complex KP1339 induces endoplasmic reticulum stress by inhibiting the GRP78 protein, bypassing traditional platinum resistance pathways (IC50 value in resistant cell lines decreased to 0.8 μM, 7 times lower than cisplatin) (Bratsos et al., 2007); while the titanium complex budotitane exerts cytotoxicity by targeting microtubule polymerization, resulting in an 80% reduction in IC50 value in cisplatin-resistant cell lines (Wang and Lippard, 2005). Recent preclinical studies have also found that iron-based complexes, such as Ferrocifen, generate reactive oxygen species (ROS) while simultaneously inhibiting the resistance-related proteins P-gp and MRP1, providing a new approach for reversing MDR (resistance reversal index of 4.2 times) (Li W. et al., 2021).

### 4.3 Stability in biological systems

Metallodrugs must remain stable and active form under physiological conditions to reach their intended targets. Many ionic metallo-drugs are prone to hydrolysis or reduction in the bloodstream, which can lead to premature deactivation or unwanted reactions with biomolecules, rendering them ineffective or toxic. The hydrolytic instability of platinum-based drugs (such as oxaliplatin) in aqueous environments limits their therapeutic efficacy (Alešković and Šekutor, 2024). A promising solution to this challenge involves ligand engineering: rigid bidentate ligands (e.g., 1,2-diaminocyclohexane) can extend the hydrolysis half-life of platinum complexes to over 24 h (CHANEY, 1995). Encapsulation of metallodrugs within stable delivery systems, such as liposomes or dendrimers, has also been explored to protect metal ions from degradation and release them in a controlled manner at the site of action. For example, liposomal cisplatin reduces blood clearance by five times and increases tumor accumulation by three times (Stathopoulos et al., 2005). Additionally, biomineralization strategies, such as biomimetic calcium phosphate coatings, can protect copper complexes in the bloodstream, releasing active components only in the acidic tumor microenvironment (Huang N. et al., 2024). Notably, metal-organic frameworks (MOFs), such as zirconium-based MOFs loaded with arsenic compounds, demonstrated a 12-fold increase in plasma stability and achieved tumor-specific accumulation via the enhanced permeability and retention (EPR) effect (Karati et al., 2024). These advances offer a theoretical foundation for designing “smart-stable” metallodrugs.

### 4.4 Limited understanding of mechanisms of action

Another fundamental challenge in using metal ions and complexes for therapeutic purposes lies in understanding their complex interactions with biomolecules. This lack of mechanistic insight impedes rational drug design and optimization efforts, particularly regarding their toxicity *in vivo*. For example, the mechanisms of newer metal-based drugs—such as ruthenium, gold, and copper complexes—are less well-understood (Meiling et al., 2013). Metal ions, such as Na^+^, K^+^, Mg^2+,^ and Ca^2+^, are essential in maintaining electrolyte balance. Activating enzyme systems. They are often found in metalloproteins with catalytic properties known as metalloenzymes. These metal ions are essential in processes such as RNA and DNA replication, positioning them as critical pharmaceutical targets (Medici et al., 2015). However, in excess, metal ions can become toxic or even carcinogenic. This toxicity results from the saturation of natural ligands and macromolecules, leading to the disruption of normal physiological processes. Thus, the difference between essential and toxic levels of metal ions is often narrow, and the dual behavior of metals at various concentrations provides the basis for threshold concentrations in carcinogenicity (Medici et al., 2015). For instance, while metals such as arsenic and antimony have long been used in traditional medicine, their therapeutic and toxic properties require careful management in modern drug formulations. Coordination chemistry in biological systems is particularly relevant in this regard, offering insights into the binding and reactivity of metal ions in proteins and enzymes (European Cooperation in Science and Technology, 2012). Recent advances in Cryo-EM (Cryogenic Electron Microscopy) technology have provided breakthroughs in elucidating the structures of metallodrug-target complexes. For example, the atomic-level structure of the ruthenium complex KP1019 binding dynamically to the RNA-binding domain of nucleolin (Kd = 12.3 nM) has been solved, revealing its selective inhibition mechanism of rRNA synthesis (Jumper et al., 2021). Furthermore, AI tools such as AlphaFold-Multimer have successfully predicted the complex structure of a palladium compound with the BRCA1 protein (RMSD = 1.2 Å), providing a new direction for designing DNA repair-targeted drugs (Gelot et al., 2023).

### 4.5 Novel model of research

It might be surprising to some that many metallodrugs on the market today are being used in patients without a thorough understanding of the active structure, behavior in the biological environment, or indeed the exact molecular mechanisms of action; the beneficial therapeutic effect of these metallodrugs is the sole reason of their continuing use in the clinic. The majority of approved metallodrugs are either quite old such as Pepto-Bismol, aurothioglucose or are, despite their toxic side effects, still in use for the treatment of a neglected disease occurring in a developing country, such as melarsoprol against human African sleeping sickness, for which advanced treatment options with less side effects have not yet been developed. This may be due to the model of research. For a long time, the research model of inorganic drugs was the same as that of organic drugs, where drug candidates were designed, synthesized, and screened on the basis of the structure of the target molecule. Only recently, attention has been paid to the study of metal ions and inorganic small molecules to intervene in the mechanism of physiopathological processes, with special attention to the differences between inorganic and organic drugs in terms of absorption, transit, distribution, metabolism, toxicology, and pharmacological effects (Bruijnincx and Sadler, 2008). At present, the research on metal drugs covers antitumor drugs, antidiabetic drugs, antiparasitic drugs, antibacterial drugs, and so on. Among them, antitumor and antidiabetic inorganic drugs are currently the main areas of interest, and major progress has been made in rational drug design. However, both have also encountered serious bottlenecks (Meiling et al., 2013). As a powerful tool for studying protein structure, an AI tool OpenFold precisely predicts more and more protein structures (Ahdritz, et al., 2024). As a result, AI tools can help us solve the problem of the metallodrug interaction targeting proteins in the future.

### 4.6 Environmental and cost concerns

The synthesis and disposal of metallodrugs can have significant environmental impacts due to the toxic nature of metal-containing waste products (Sheldon, 2017). In addition, the high cost of some precious metal-based drugs, such as those involving platinum and gold, can make them inaccessible for widespread use in clinical settings. To address these challenges, green chemistry and alternative metal strategies are rapidly developing. Engineered *Escherichia coli* can recover 98% of platinum from waste liquids, reducing costs by 70% compared to traditional methods, providing a sustainable solution for the recycling of precious metals (Tan et al., 2020). Microwave-assisted aqueous-phase synthesis of iron-based nanoparticles (Fe3O4@ZIF-8) can reduce energy consumption by 85% and completely avoid the use of organic solvents (Yatoo et al., 2023). In addition, addressing these issues also requires developing more sustainable and cost-effective metallodrugs. Researchers are exploring the use of more abundant and environmentally benign metals, such as iron, copper, and zinc, as alternatives to precious metals. Moreover, green chemistry approaches aimed at reducing the environmental footprint of metallodrug synthesis are being actively developed, such as using aqueous-phase reactions and recyclable catalysts.

## 5 Conclusion and future directions

The development of metallodrugs presents multiple challenges, including toxicity, drug resistance, stability, limited mechanistic understanding, and environmental concerns. However, advances in targeted drug delivery, combination therapiesand green chemistry offer promising avenues to resolve these issues. Metallodrugs will become a hot topic in medical research and modern medicine development in the future, once these challenges are addressed.

In diagnostic medicine, metallodrugs drugs, particularly gadolinium and technetium, enhance imaging technologies such as magnetic resonance imaging (MRI) and radioactive imaging due to their unique properties as paramagnetic and radioactive labeling agents, respectively (Kanal, 2016). The incorporation of metals into drug design not only expands therapeutic options but also introduces new challenges in understanding their interactions with biological systems (Boros et al., 2020).

Looking ahead, medicinal inorganic chemistry demonstrates tremendous potential, with ongoing research focused on the kinetics and thermodynamics of metal interactions within biological environments. This research is crucial for developing novel metallodrugs that can both enhance therapeutic efficacy and minimize side effects. Current studies are exploring the application of transition metals in bimodal imaging and targeted therapy (Ko et al., 2019). By integrating inorganic chemistry with medical applications, metallodrugs have the potential to address major health concerns, positioning them as a critical component of modern therapeutic strategies.

Historically, inorganic drugs have played a significant role in chemotherapy, with metals such as arsenic used in the treatment of microbes, parasites, and cancer. Despite a reduction in their use with the emergence of organic drugs, due to concerns over toxicity and limited therapeutic advantages, inorganic drugs experienced a resurgence in the late 20th century. Notably, arsenic trioxide’s success in treating acute promyelocytic leukemia highlighted the continued exploration of inorganic compounds, such as gold and copper complexes, for their anti-inflammatory and antiviral properties (Medici et al., 2015).

While significant progress has been made in inorganic drug research, challenges remain. Given the unique properties of inorganic drugs, future research must continue to focus on achieving a balance between therapeutic effects and toxicity, developing suitable drug formulations, and establishing proper standards for the stability, composition, and quality control of these compounds. Future research should concentrate on the following areas: 1) discovering new molecular mechanisms of action for metal-based drugs; 2) controlling the toxicity of metal-based drugs; 3) utilizing nanotechnology and molecular complexes to develop novel drug delivery systems (Ahmed et al., 2024; Bertrand et al., 2014); 4) advancing the field of synthetic biology for metal complexes. These areas are key to overcoming current limitations and maximizing the therapeutic potential of metal-based drugs.
